# Title: Reducing APOE4 in carriers is a therapeutic goal for Alzheimer Disease: Report of the APOE4 National Institute on Aging (NIA) /Alzheimer Disease Sequencing Project (ADSP) Consortium Working Group

**DOI:** 10.1002/alz.093142

**Published:** 2025-01-03

**Authors:** Jeffery M. Vance, David M. Holtzman

**Affiliations:** ^1^ John P. Hussman Institute for Human Genomics, Miami, FL USA; ^2^ University of Miami Miller School of Medicine, Miami, FL USA; ^3^ Dr. John T. Macdonald Foundation Department of Human Genetics, University of Miami Miller School of Medicine, Miami, FL USA; ^4^ Washington University School of Medicine in St Louis, St. Louis, MO USA

## Abstract

As part of the goal of the ADSP to develop effect therapeutic for AD, it has become imperative to develop therapeutic targets for APOE4. The Apolipoprotein E4 gene (APOE4) strongest genetic risk factor for AD and is present in 64% of sporadic, late‐onset AD, with a recent prevalence estimate of 245 million carriers worldwide. A key question is whether APOE4 is a toxic or gain of function allele or alternatively if its effect is due to a loss or partial loss of function.

To answer this question, the APOE4 National Institute on Aging (NIA)/Alzheimer’s Disease Sequencing Project Consortium (ADSP) working group (WG) came together to discuss the question of whether to reduce or increase APOE4 as therapeutic intervention for AD. The WG reviewed both genetic data concerning this question and the various model studies on the effects of reducing or increasing the expression of APOE4.

Studies utilizing ancestry comparisons, single nuclei RNAseq/ATACseq of AD autopsy material and studies of loss of function variants in APOE all provided support that the appropriate therapeutic intervention for APOE4 carriers is to reduce APOE4 expression and APOE4 protein levels. Reviewing studies in animal and cellular models to assess the effects of raising or lowering APOE4 conclusively demonstrated that the presence of APOE4 leads to a toxic gain of function and that lowering it’s levels in the brain is the therapeutic choice. The WG only reviewed African and European ancestries as genomic data is from Asian ancestry is limited.

It was the unanimous conclusion by the WG that lowering APOE4 is the correct therapeutic intervention. The combined studies strongly support that APOE4 exerts a toxic gain of function in regard to not only risk for AD but also on several outcomes linked with AD including amyloid deposition, neurodegeneration that is tau‐mediated and inflammation. Questions that are important to address as intervention moves forward include: What would be the timing and duration of therapy? How does local ancestry influence therapeutic options? Given that APOE is needed for normal physiological function, how far should it be lowered? How would successful outcome be monitored?

